# Neural correlates underlying state anxiety alterations following sleep deprivation: insights from frontal alpha asymmetry and phase-amplitude coupling

**DOI:** 10.3389/fpsyg.2025.1633875

**Published:** 2025-07-11

**Authors:** Chao Hao, Feiyang Xie, Naifeng Bu

**Affiliations:** Research Center for Social and Economic Development of Comprehensive Health and Aging, School of Art and Design, Guangzhou College of Commerce, Guangzhou, China

**Keywords:** sleep deprivation, state anxiety, resting-state EEG, frontal alpha asymmetry, delta-beta phase-amplitude coupling

## Abstract

**Introduction:**

Elevated anxiety stands as a prominent adverse consequence of sleep deprivation. However, the resting-state neural correlates underlying the alterations in state anxiety following sleep deprivation remain to be further examined. The present study delved into the impact of sleep deprivation on state anxiety and its underlying neural correlates.

**Methods:**

With 20 participants, we recorded their resting-state electroencephalogram activities and assessed their state anxiety using the State Anxiety Inventory Scale during both normal sleep and 24-hour sleep deprivation sessions.

**Results:**

Results show that sleep deprivation induced a significant increase in state anxiety. Additionally, there was a general elevation in resting-state electroencephalographic power, with notable increases observed in the delta, theta, alpha, and beta frequency bands, as well as the delta-beta phase-amplitude coupling. Meanwhile, the frontal alpha asymmetry presented a left lateralization after sleep deprivation. Furthermore, the heightened beta2 power and delta-beta2 phase-amplitude coupling exhibited a significant positive correlation with increased state anxiety after sleep deprivation. The left-lateralized frontal alpha asymmetry was linked to the enhancement in state anxiety following sleep deprivation.

**Discussion:**

The present study provided the resting-state electroencephalographic evidence that accounts for the magnified state anxiety after sleep loss.

## 1 Introduction

Sleep serves as a dynamic physiological process that is indispensable for human survival, typically accounting for roughly one-third of our lifespan (Simon et al., 2022; Garbarino et al., 2021). It is important in sustaining our physical, mental, and cognitive health. For adults, the importance of adequate sleep is underscored by numerous scientific studies. A minimum of 7 h of sleep per night is widely regarded as the gold standard for achieving and maintaining optimal health (Gallicchio and Kalesan, 2009; Li et al., 2022). However, in the fast-paced and highly demanding modern societies of today, there is a growing tendency for individuals to sacrifice sleep in favor of work, social activities, or entertainment. As a result, many people are failing to meet the recommended number of nocturnal sleep hours. The constant bombardment of stimuli from technology, the increasing prevalence of shift work, and the high-stress lifestyles that have become the norm are all contributing factors to this sleep-loss state.

Additionally, anxiety stands as the most prevalent mental health problem globally (Saxena et al., 2014), which is an inner biological reaction marked by feelings of fear and heightened vigilance to potential dangers or uncertain threats (Leal et al., 2017). There are two types of anxiety: trait anxiety and state anxiety. State anxiety is a temporary psychological response to unfavorable circumstances, while trait anxiety is a persistent mood state related to personality characteristic (Spielberger et al., 1971). Epidemiological studies show that insufficient sleep could trigger or further intensify state anxiety (Chellappa and Aeschbach, 2022). Sleep deprivation serves as a potent experimental tool to elucidate the connections between sleep and anxiety. Elevated anxiety is one of the most important negative effects of sleep deprivation (Pires et al., 2016).

Numerous studies have attempted to explain the alternations in neural activity related to anxiety after sleep deprivation. Task-related studies have demonstrated elevated anxiety is related to increased activation of the insula after sleep deprivation (Ben Simon et al., 2020; Krause et al., 2017). However, state anxiety typically emerges in the absence of an identifiable stimulus. Indeed, the human brain remains actively engaged in spontaneous neural activities even in the absence of external stimuli (Raichle and Mintun, 2006; Zhang et al., 2019). Resting-state EEG is a valuable tool for investigating these spontaneous activities due to its high time resolution and non-invasive nature, enabling direct measurement of cortical functioning (Rossini et al., 2007). Several studies have proven that the resting-state EEG power increased from delta to beta bands after sleep deprivation (Gorgoni et al., 2014; Hao et al., 2023). Increased power within each frequency range exhibited diverse behavioral correlations. Higher delta, theta, and alpha activity during wakefulness were related to increased sleep propensity and decreased vigilance after sleep deprivation. At the same time, motivation-related research further implicated the role of delta oscillations in reward processing and alpha rhythms in anxiety regulation (Knyazev et al., 2006). In addition, enhanced beta activity might reflect the hypervigilance and rumination that occur even in the absence of external stimuli (de Carvalho et al., 2015; Levy et al., 2025). The specific relationship between alterations in resting-state EEG spectral activities and corresponding changes in state anxiety following sleep deprivation remains to be further examined.

Furthermore, resting-state frontal alpha asymmetry has long been applied in the study of state anxiety (Akil et al., 2024; Coan and Allen, 2004; Monni et al., 2022). A study found marginally more left-lateralized frontal alpha power after sleep deprivation (Zhang et al., 2019). Alpha bands’ activity in a specific area inversely correlates with its actual cortical activation levels (Barry et al., 2007; Zhang et al., 2019). Heightened alpha power signifies relative neural deactivation, whereas reduced alpha power indicates relatively increased neural activation. Moreover, the different hemispheres of the frontal lobe play distinct functional roles. The left hemisphere is often linked to positive affect, referred to as the “happy” brain, whereas the right hemisphere is commonly associated with negative affect, termed the “sad” brain (Zhang et al., 2019). Consequently, left lateralization of alpha power has been linked to anxiety disorders. Inversely, the right lateralization of alpha power reflects stronger emotion regulation. The link between sleep deprivation-induced changes in resting-state frontal alpha asymmetry and subsequent state anxiety modulation requires further investigation.

Beyond spectral power analysis of brain oscillations, cross-frequency coupling (CFC) has emerged as a critical mechanism for decoding the neurodynamic architecture of anxiety-related processes. CFC offers a promising way of understanding the intricate interactions of diverse information-processing mechanisms within the brain (Hyafil et al., 2015; Stankovski et al., 2017). Phase-amplitude coupling (PAC) is a common type of CFC, reflecting the coupling between the phase of low frequency and the amplitude of high frequency (Gwon and Ahn, 2021). High-frequency oscillations enhance local neural coordination, while low-frequency oscillations facilitate longer-distance communication (Popov et al., 2012; Riddle et al., 2022). Delta-beta coupling has been indicated as one of the neural correlates of emotion regulation (Knyazev et al., 2006; Schutter and Knyazev, 2012). Converging evidence has suggested increased delta-beta coupling during state anxiety and under situations of uncertainty (Jaiswal et al., 2023; Knyazev et al., 2005). However, it remains unclear the impact of sleep deprivation on delta-beta coupling, and the relationship between this coupling and altered state anxiety after sleep deprivation.

The present study aimed to explore the impact of sleep deprivation on state anxiety and resting-state neural activities, as well as the neural correlates underlying state anxiety alterations following sleep deprivation. Utilizing a between-group design, we compared the state anxiety, the resting-state power spectrum, the frontal alpha asymmetry, and the delta-beta PAC between sleep deprivation and normal sleep sessions. Subsequently, we explored whether the altered state anxiety observed after sleep deprivation was linked to the changes in the spectral power, the frontal alpha asymmetry, and the delta-beta PAC. It was hypothesized that sleep deprivation would lead to an increase in state anxiety. Furthermore, it was anticipated that the resting-state power spectrum in various frequency bands, including delta, theta, alpha, and beta, would increase after sleep deprivation (Liu et al., 2024). Meanwhile, the frontal alpha asymmetry score would decrease (left lateralization of alpha power), and the delta-beta PAC would increase (Zhang et al., 2019). Finally, the increased delta-beta PAC and decreased frontal alpha asymmetry score were linked to increased state anxiety after sleep deprivation.

## 2 Methods

### 2.1 Participants

The current study enrolled 20 healthy adults and was conducted in the Center for Sleep Research at the South China Normal University. Three participants were deleted due to insufficient epochs (less than 80%) caused by excessive artifacts in EEG recording. The final analysis sample contained 17 participants (19.24 ± 1.20 years old; 11 females, 6 males). All participants signed written informed consent and received financial compensation for their time following their participation.

Participants were recruited based on a structured screening questionnaire. Eligibility criteria included: (1) no history of medical, neurological, psychiatric, or sleep-related disorders; (2) no caffeine or alcohol addiction; (3) had no any symptoms of depression or anxiety, defined as having a Beck Depression Inventory score < 10 and a State–Trait Anxiety Inventory score < 53 for males and 55 for females; (4) had good sleep quality over the past months, defined as having a Pittsburgh Sleep Quality Index score ≤5 (Buysse et al., 1989).

### 2.2 Procedures

**Figure 1:**
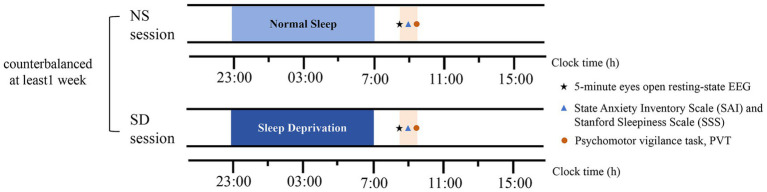
Study protocol. Asterisks denote a 5-min eyes-open resting-state EEG recording. Triangles denote the State Anxiety Inventory Scale (SAI) and the Stanford Sleepiness Scale (SSS). Circles denote the Psychomotor vigilance task (PVT). NS represents normal sleep, SD represents sleep deprivation.

One week prior to the experiment, participants were instructed to maintain a habitual sleep duration of 7–8 h and adhere to a bedtime of 23:00 ± 1 h nightly. Sleep quality and quantity were assessed using sleep diaries and wrist actigraphy (Actiwatch Spectrum, Philips). All participants completed two laboratory sessions: a normal sleep (NS) protocol and a sleep deprivation (SD) protocol, as illustrated in Figure 1. The order of these sessions was counterbalanced across participants, with at least one week separating them. To fully eliminate residual effects of SD, the NS session was conducted one month after the SD session. During this washout period, participants were required to maintain a consistent daily sleep–wake schedule, as outlined above. In the NS session, participants slept in their dormitories according to their habitual bedtime (23:00–07:00). The following morning, they underwent a 5-min resting-state EEG recording with eyes open in the laboratory. This condition was chosen to better approximate the cognitive state during task performance compared to eyes-closed conditions. Participants were instructed to fix on a white cross presented on a black screen and to avoid eye movements or specific thoughts. After the resting-state EEG recording, they completed the State Anxiety Inventory (SAI). To validate the experimental manipulation, participants also performed a 5-min psychomotor vigilance task (PVT) and completed the Stanford Sleepiness Scale (SSS). The SSS and PVT served as subjective and objective measures of sleepiness, respectively. In the SD session, participants underwent 24 h of total sleep deprivation, with continuous monitoring by trained experimenters to ensure they remained awake. The following day, the 5-min eyes-open resting-state EEG, SAI, PVT, and SSS were administered at the same time of day as in the NS session.

State anxiety was assessed utilizing the State Anxiety Inventory (SAI), a 20-item self-report measure from the State–Trait Anxiety Inventory (Spielberger et al., 1971). Participants were asked to rate the intensity of their current anxiety feelings on a 4-point Likert scale (1 = absolutely not to 4 = very much), ranging from 1 (“absolutely not”) to 4 (“very much”). The total score, spanning from 20 to 80, was used to quantify state anxiety, with higher scores indicating a greater degree of state anxiety. Subjective sleepiness was assessed using the Stanford Sleepiness Scale (SSS) (Hoddes et al., 1972), a single-item visual analogue scale. Participants rated their sleepiness on a 7-point scale (1 = feeling energetic, vital, alert, and awake to 7 = always dreaming, falling asleep fast, giving up staying awake).

Psychomotor vigilance task (PVT) is a simple reaction time task that requires participants to respond as quickly as possible to stimuli presented at random intervals (Shenfield et al., 2020). In this study, a 5-min PVT protocol was employed. Participants monitored a rectangular box on a computer screen and pressed a response button immediately upon the appearance of a red number. Valid responses were those with reaction times (RT) ≥ 100 ms, while lapses were defined as RT ≥ 500 ms. The number of lapses and mean RT were selected as the primary PVT indices.

### 2.3 Electroencephalography (EEG) recording and processing

EEG data were recorded at a sampling rate of 500 Hz using 64 Ag/AgCl electrodes mounted in an electrode cap, referenced to FCz. Recordings were conducted with Brain Vision Recorder (Brain Products GmbH, Gilching, Germany), with electrode impedance maintained below 5 kΩ. Offline data analysis was performed using MATLAB R2021b (MathWorks, Natick, MA) with EEGLAB (v2020.0) and custom scripts leveraging EEGLAB functions. Data were re-referenced to the average reference. Noisy channels were visually identified and interpolated using spherical spline interpolation. All signals were filtered with a 0.1 Hz high-pass filter, an 80 Hz low-pass filter, and a 50 Hz notch filter to remove power-line noise. Stereotyped artifacts (e.g., blinks, vertical eye movements, and muscle activity) were removed using a semi-automatic Independent Component Analysis (ICA)-based procedure. The cleaned continuous EEG during the eyes-open resting-state was then segmented into 2 s epochs, yielding a frequency resolution of 0.5 Hz. Epochs containing non-stereotyped artifacts (e.g., cable movement, swallowing) or signals exceeding ±150 μV were excluded from further analysis. For NS session, an average of 97.02% of components and 96.53% of epochs were retained. For SD session, the retention rates were 97.49% of components and 93.69% of epochs.

### 2.4 Spectral analysis, frontal alpha asymmetry, and PAC

Eyes open resting-state EEG data was analyzed using spectral analysis performed using custom scripts based on FieldTrip. Power spectral distributions of single-epoch EEG signals were estimated using a MTMFFT (multi-taper-method Fast Fourier Transform) with a fixed Hanning taper. Frequency points ranged from 1 to 80 Hz. The power spectrum was averaged across the range of each of the six frequency bands (delta: 1–4 Hz; theta: 4–7 Hz; alpha: 8–13 Hz; beta1: 13–20 Hz, beta2: 20–30 Hz with 10 Hz steps).

Frontal alpha asymmetry was determined by calculating the difference between the natural logarithm of alpha power at the right frontal and the natural logarithm of alpha power at the left frontal. Extensive research has demonstrated a consistent link between emotional functions and alpha asymmetry at the F3 and F4 electrodes (Zhang et al., 2020; Zhang et al., 2019). In addition, the test–retest reliability of frontal alpha EEG recordings at the F3-F4 electrode pair has been demonstrated to achieve acceptable standards (Winegust et al., 2014). We computed frontal alpha asymmetry by subtracting the natural logarithm of alpha power at the F3 electrode site from that at the F4 electrode site (specifically, ln[F4 alpha]−ln[F3 alpha]).

PAC analysis was run between the phase of delta oscillations (1–4 Hz) and the amplitude of beta oscillations (beta1:13-20 Hz, beta2:20-30 Hz) in every channel across the scalp. PAC was computed using the mean vector length modulation index (MVL-MI) described by (Canolty et al., 2006). The phase of delta and the amplitude of beta derived from the Hilbert transform of band-filtered data using function “eegfilt” in EEGLAB. For each participant, phase (*θ*) and amplitude (M) values of each epoch were concatenated into a single continuous time series (n is the number of time points) and PAC was calculated according to Equation 1. Then, surrogate tests were conducted by randomly partitioning the signal into 2 slices and rearranging the order of these slices. After 1,000 repetitions, PAC values were converted to normalized PAC_Z_. Genuine PAC can only be positive. Thus, whether PAC was present in a single condition was investigated using one-tailed tests.


(1) $$\mathrm{PAC}=|\frac{\sum_{t=1}^{n}M*e^{\mathrm{i\theta}}}{n}|$$

### 2.5 Statistics

Statistical differences in SAI, SSS, PVT indices, and the frontal alpha asymmetry between NS and SD sessions were tested by 2-tailed paired *t*-tests. To explore how sleep deprivation modulated the topographic electroencephalogram changes and the association with state anxiety, all channels were selected for further analysis. The power spectrum in the six frequency bands (delta, theta, alpha, beta1, beta2), and the delta-beta PAC were analyzed by 2-tailed paired *t*-tests in each electrode separately. Moreover, Pearson’s correlations were employed to evaluate the relationships between the change (sleep deprivation–normal sleep) of SAI and electrophysiological data (the power spectrum, PAC in each electrode, and the alpha asymmetry). Analyses were conducted using SPSS 23.0, with statistical significance set at *p* < 0.05 (two-tailed) for all tests. To address multiple comparisons across electrode pairs, the False Discovery Rate (FDR) correction was applied, and corrected *p*-values are reported.

## 3 Results

### 3.1 State anxiety

**Figure 2:**
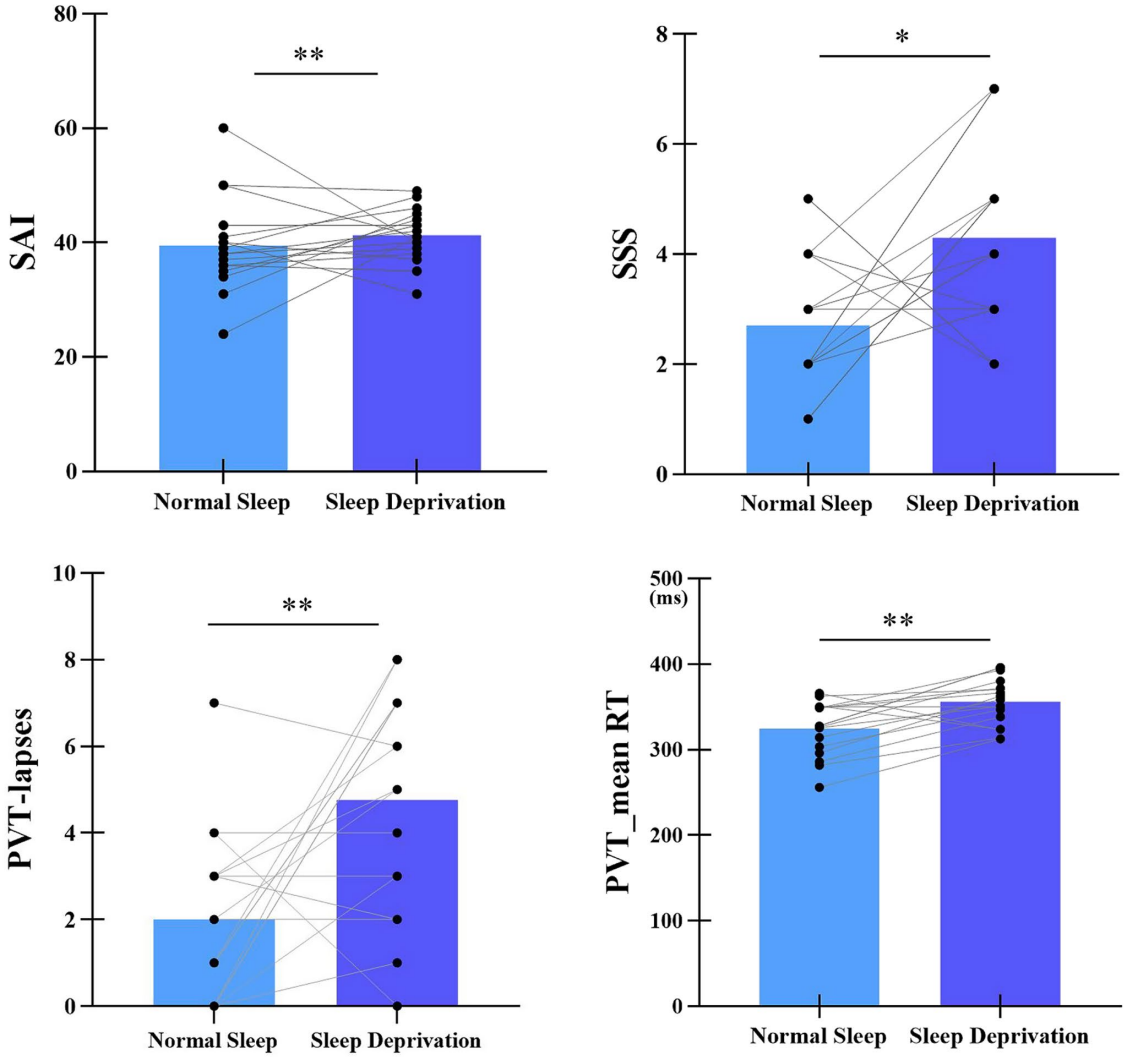
Statistical differences in SAI, SSS, mean RT, and number of lapses between normal sleep and sleep deprivation sessions. SAI represents State Anxiety Inventory, SSS represents Stanford Sleepiness Scale. **p < 0.01, *p < 0.05.

The statistical differences in SAI, SSS, mean RT, and number of lapses between normal sleep and sleep deprivation sessions were listed in Figure 2. Compared with normal sleep session, state anxiety significantly increased in the sleep deprivation session (*t*_(16)_ = −2.95, *p* < 0.01, Cohen’*d* = 0.59). In addition, sleep deprivation significantly increased the number of lapses (*t*_(16)_ = −3.2, *p* < 0.01, Cohen’*d* = 0.62), mean RT (*t*_(16)_ = −3.98, *p* < 0.01, Cohen’*d* = 0.71), and sleepiness (*t*_(16)_ = −2.50, *p* < 0.05, Cohen’*d* = 0.53).

### 3.2 Changes in resting EEG power and the relationship with SAI after SD

**Figure 3:**
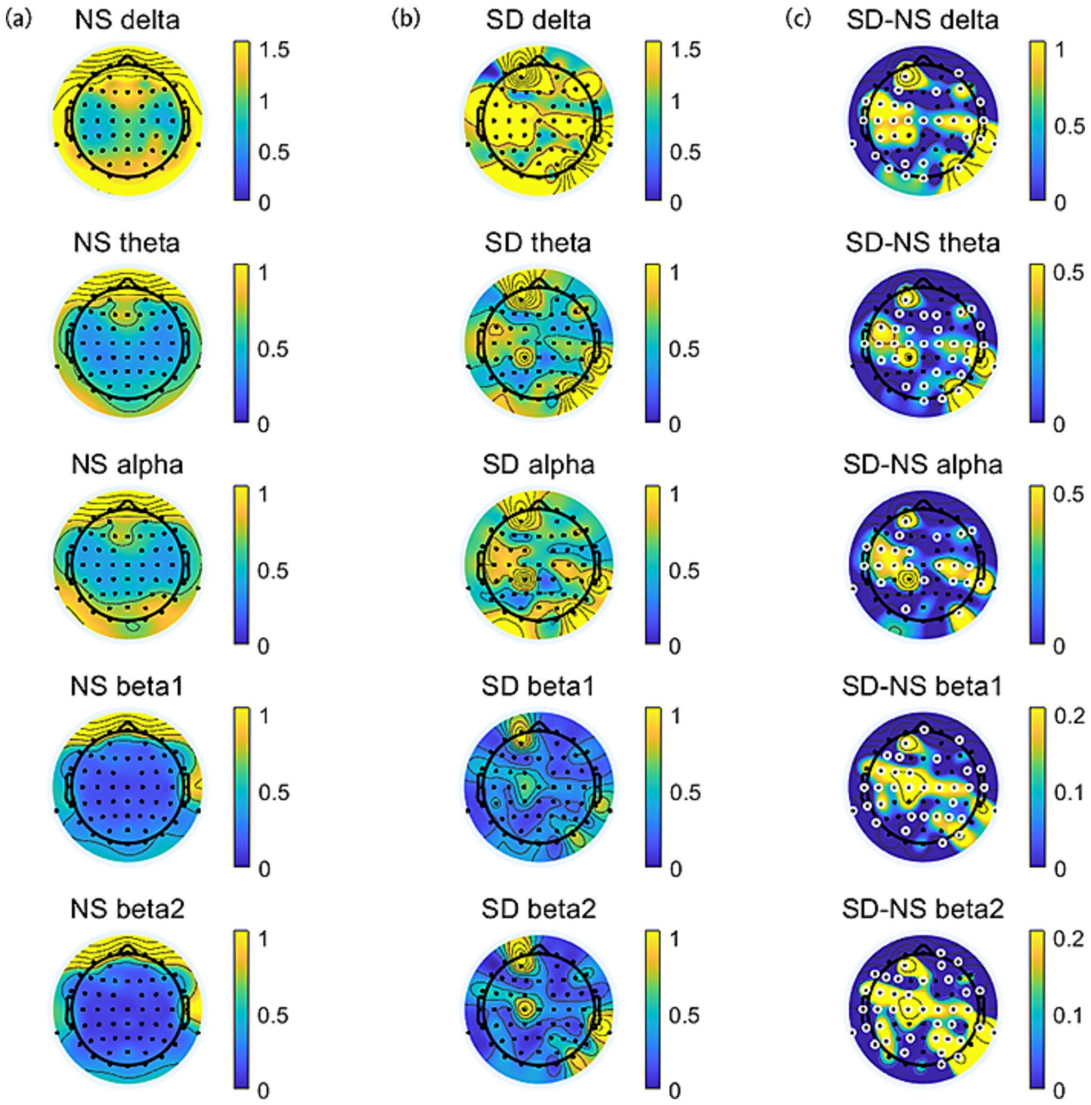
The topographic distribution of spectral power across frequency bands (delta, theta, alpha, beta1, and beta2). **(a)** Normal sleep session. **(b)** Sleep deprivation session. **(c)** The difference (SD–NS). Electrodes with significant differences between sessions are highlighted by enlarged white circles.

The topographic distribution of spectral power during normal sleep (NS) and sleep deprivation (SD) sessions was illustrated in Figure 3. A significant difference in EEG power spectra was observed between the two sessions. Specifically, the SD session exhibited a generalized increase in delta, theta, alpha, and beta power compared to the NS session (all *p* < 0.05, FDR corrected).

**Figure 4:**
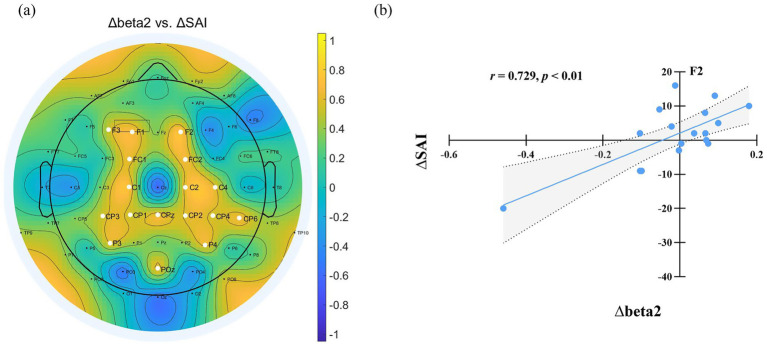
**(a)** Relationship between EEG power changes and SAI variation after sleep deprivation. ∆ denotes the difference in EEG power and State Anxiety Inventory (SAI) scores between the sleep deprivation (SD) and normal sleep (NS) sessions (SD–NS). Electrodes showing a significant correlation between changes in EEG power and SAI variation are highlighted by enlarged white circles. **(b)** Changes in beta2 power at F2 were significantly positively correlated with the changes in state anxiety across the two sessions.

The differences in State Anxiety Inventory (SAI) scores and EEG power spectra between the normal sleep (NS) and sleep deprivation (SD) sessions were denoted as ∆SAI and ∆EEG power (∆delta, ∆theta, ∆alpha, ∆beta1, and ∆beta2), respectively. Correlation analyses were conducted to examine the relationships between ∆SAI and ∆EEG power. A significant positive correlation was observed between ∆SAI and ∆beta2 power (SD–NS), indicating that greater increases in beta2 power after sleep deprivation were associated with higher state anxiety. This correlation was primarily localized to electrodes in the frontal-central region (F1, F2, F3, FC1, FC2, C1, C2, C4, CPZ, CP1, CP2, CP3, CP4, CP6, P3, P4, POZ; FDR-corrected), as illustrated in Figure 4.

### 3.3 Changes in frontal alpha asymmetry and the relationship with SAI after SD

**Figure 5:**
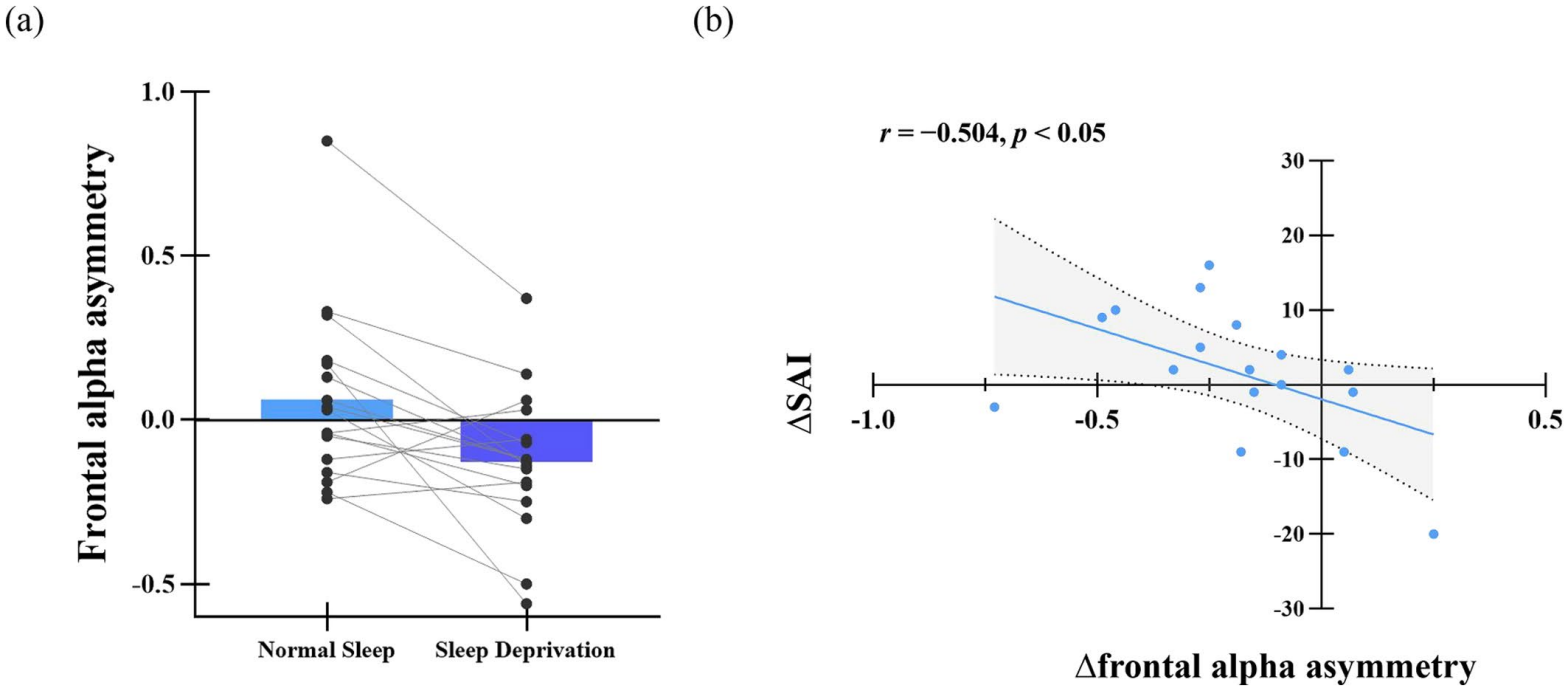
**(a)** Difference in frontal alpha asymmetry score. **(b)** Relationship between frontal alpha asymmetry change and SAI variation. *Δ* denotes the difference in frontal alpha asymmetry scores between the sleep deprivation (SD) and normal sleep (NS) sessions (SD–NS).

Figure 5a illustrates frontal alpha asymmetry scores for the normal sleep (NS) and sleep deprivation (SD) sessions. A paired-*t* test revealed a significantly higher asymmetry score in the NS session (*M* = 0.06, *SD* = 0.27) compared to the SD session (*M* = −0.13, *SD* = 0.22; *t*_(16)_ = 3.32, *p* < 0.01, Cohen’s *d* = 0.64). Further correlation analysis indicated a negative relationship between ∆SAI (change in state anxiety: SD–NS) and ∆frontal alpha asymmetry (SD–NS; *r* = −0.504, *p* < 0.05), as shown in Figure 5b. This suggests that greater left-lateralized alpha activity (lower asymmetry scores) is associated with higher state anxiety following sleep deprivation.

### 3.4 Changes in delta-beta PAC and the relationship with SAI after SD

**Figure 6:**
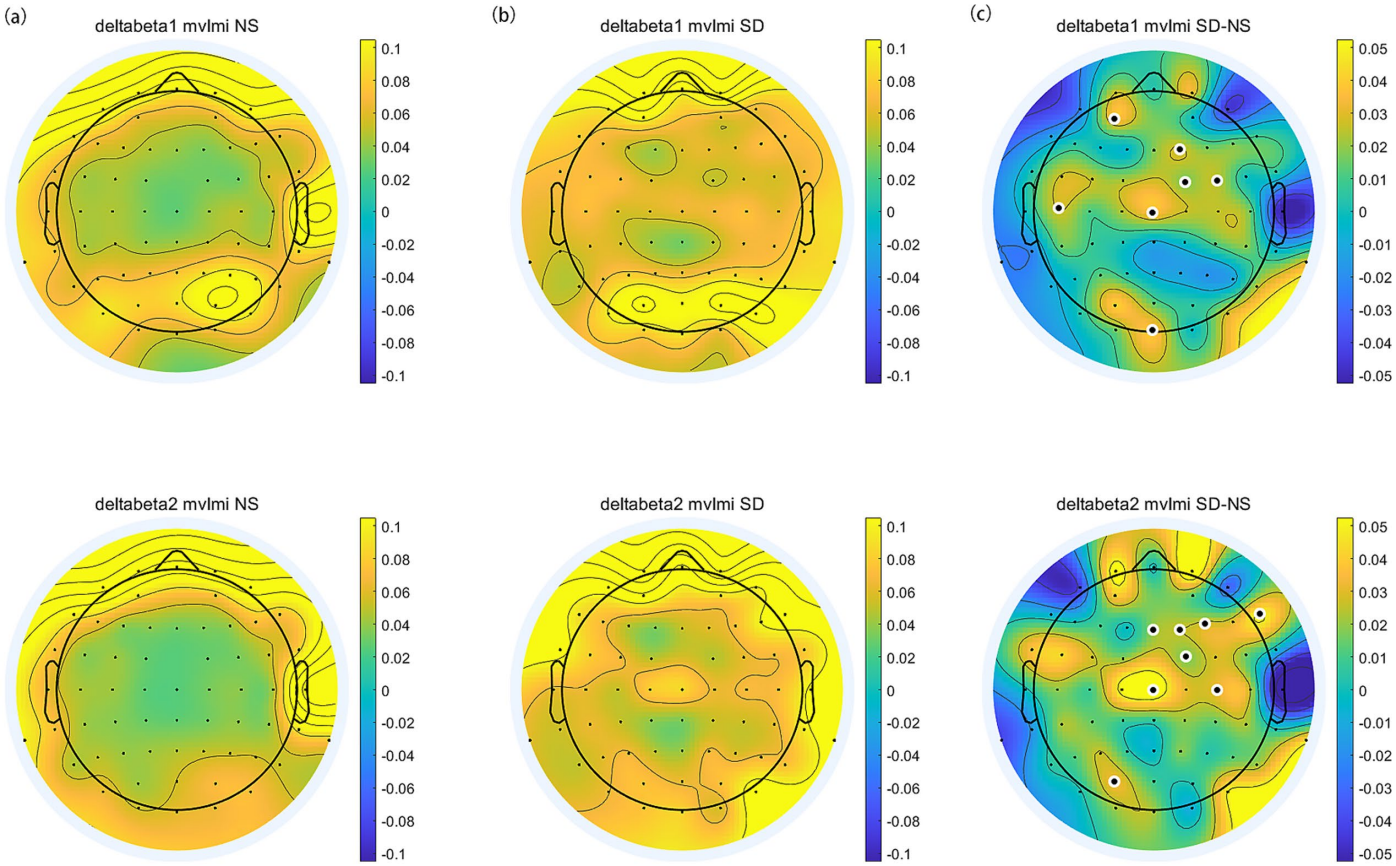
The topographic distribution of delta-beta PAC. **(a)** Normal Sleep session. **(b)** Sleep Deprivation session. **(c)** The difference (SD–NS). Electrodes with significant differences between sessions are highlighted by enlarged white circles.

**Figure 7:**
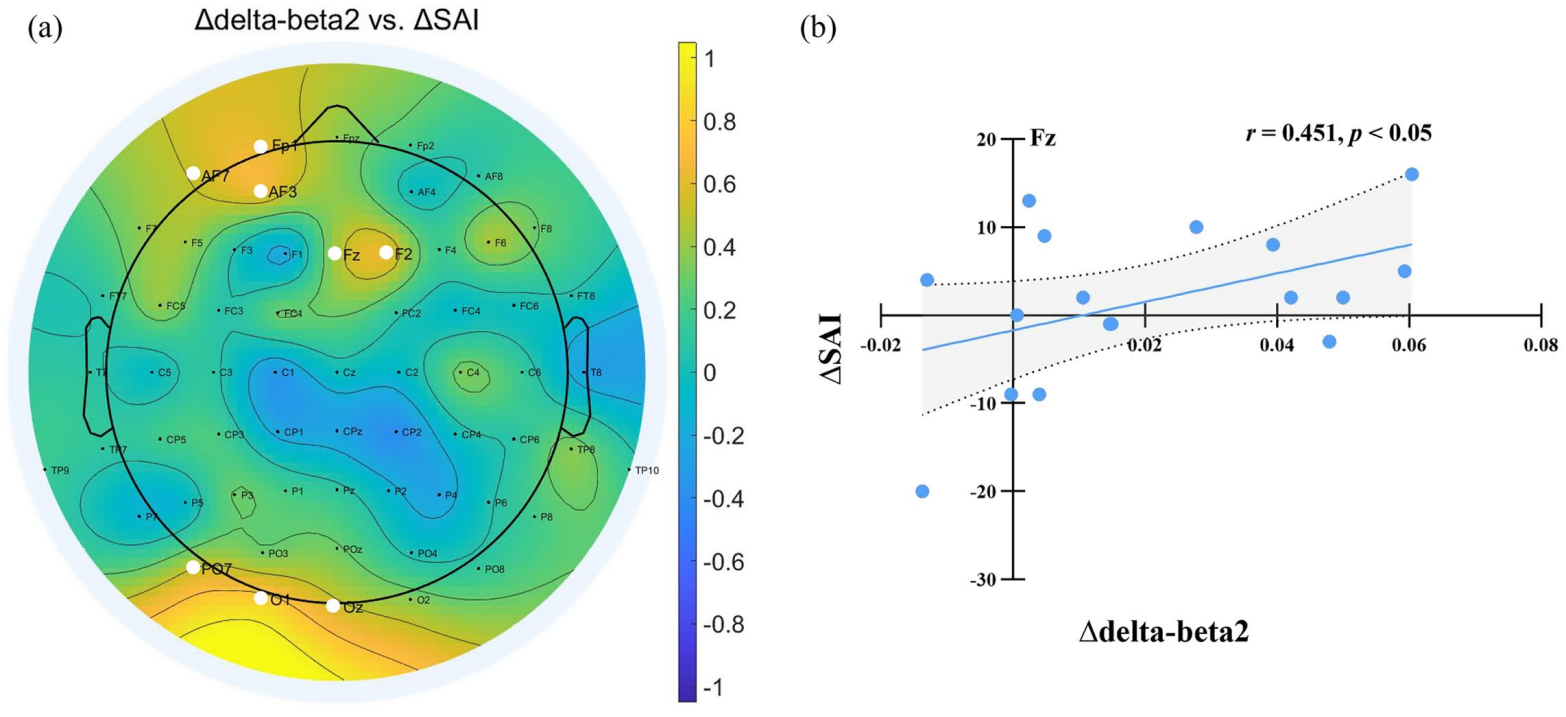
**(a)** Relationship Between delta-beta PAC Changes and SAI Variation After Sleep Deprivation. ∆ denotes the difference in EEG power and State Anxiety Inventory (SAI) scores between the sleep deprivation (SD) and normal sleep (NS) sessions (SD–NS). Electrodes showing a significant correlation between changes in delta-beta PAC and SAI variation are highlighted by enlarged white circles. **(b)** Changes in delta-beta2 PAC power at Fz were significantly positively correlated with the changes in state anxiety across the two sessions.

We estimated PAC using the delta phase to the amplitude of beta oscillations across the scalp. Delta-beta coupling was present for both the normal sleep and sleep deprivation conditions (Figures 6a,b). Furthermore, the delta-beta2 PAC in the central region increased significantly after sleep deprivation (all *p* < 0.05, FDR corrected), as shown in Figure 6c. The further correlation analysis found that ∆SAI was positively correlated with ∆delta-beta2 coupling in the frontal and occipital region (FP1, Fz, F2, AF3, AF7, PO7, Oz, and O1; all *p* < 0.05, FDR corrected), as shown in Figure 7. The more frontal and occipital delta-beta2 PAC increased after sleep deprivation, the more state anxiety increased.

## 4 Discussion

The current study found that sleep deprivation significantly increased state anxiety. Sleep deprivation led to an overall increase in the power of resting-state brain oscillations, with notable increases observed in the delta, theta, alpha, and beta frequency bands. Meanwhile, the frontal alpha asymmetry score decreased and delta-beta PAC increased significantly after sleep deprivation. In addition, the heightened beta power exhibited a significant positive correlation with increased state anxiety after sleep deprivation. The decrement in frontal alpha asymmetry score negatively correlated with the enhancement in state anxiety following sleep deprivation. Moreover, the enhancement in delta-beta2 coupling in the frontal and occipital regions positively correlated with the increase in state anxiety following sleep deprivation.

During sleep deprivation, the homeostatic process accumulates sleep pressure progressively as the duration of wakefulness increases (Cajochen et al., 2010). Consistent with prior research, sleep deprivation resulted in an overall augmentation of resting-state brain oscillation power, particularly within the delta, theta, alpha, and beta frequency bands (Gorgoni et al., 2014; Hao et al., 2023; Liu et al., 2024). This overall enhancement of spontaneous EEG power, driven by homeostatic sleep pressure, was likely associated with heightened cortical excitability, which is a sign of cortical de-arousal or cortical idling (Boonstra et al., 2007). Notably, similar spectral power augmentation patterns have been documented in highly anxious individuals facing uncertain conditions (Knyazev et al., 2005). This parallel suggests that sleep deprivation may induce a spectral state resembling anxiety-related over-rumination. The elevated spectral power observed in sleep-deprived individuals could facilitate enhanced environmental monitoring and preparatory responses, mirroring the adaptive mechanisms employed by anxious subjects when anticipating uncertain events.

The present study revealed a significant correlation between a widespread increase in beta2 power and increased state anxiety after sleep deprivation. Enhanced beta activity might reflect cognitive inflexibility, excessive rumination and over-stimulation following sleep deprivation (de Carvalho et al., 2015; Levy et al., 2025). In addition, a study revealed insular beta activity is a biomarker of state anxiety (Lee et al., 2024). The anterior insula has been recognized as a crucial component within an anxiety-related neural network (Haufler et al., 2022; Paulus and Stein, 2006). Insula beta activity is capable of detecting and anticipating potential threats, aversive stimuli, and other relevant information of anxiety (Lee et al., 2024). A large number of studies have found sleep deprivation could increase the activation of insula (Ben Simon et al., 2020; Krause et al., 2017). As a result, the elevated beta activity might reflect that sleep deprivation amplified the insula’s sensitivity to anxiety-related information. This heightened sensitivity could trigger excessive rumination, and overstimulation, ultimately resulting in an elevation of self-reported state anxiety levels.

Furthermore, the present study observed enhanced delta-beta PAC following sleep deprivation, with significant positive correlations between delta-beta2 coupling in frontal and occipital regions and state anxiety severity. Delta-beta coupling might indicate the integration between local cortical and distal limbic system regions to achieve emotion regulation (Jaiswal et al., 2023; Putman, 2011). Meanwhile, Putman (2011) used the dot-probe task to reveal that delta-beta coherence was related to selective attention to threat. High-frequency beta oscillation might pay attention to potential threats, while low-frequency delta oscillation facilitates longer-distance communication. As a result, sleep deprivation may amplify threat detection sensitivity through enhanced delta-beta synchronization, leading to increased state anxiety.

Consistent with previous studies, the present study revealed a significant left lateralization in frontal alpha asymmetry following 24-h sleep deprivation (Ferreira et al., 2006; Zhang et al., 2019). Moreover, left frontal alpha lateralization was negatively related to elevated state anxiety. That is, an enhanced dominance of right prefrontal alpha activity relative to the left was linked to an augmentation of state anxiety levels. The human brain exists in two fundamental motivational systems: the approach system, which actuates approach behaviors toward rewarding information, and the withdrawal system, which governs avoidance behaviors in response to aversive stimuli (Gray, 1990, 2014). The motivational system is closely related to the hemispheric specialization within the dorsolateral prefrontal cortex (Botvinick and Braver, 2015; Spielberg et al., 2012). Heightened activity within the right prefrontal regions, compared to the left side (left lateralization), is associated with withdrawal behaviors, whereas the converse pattern (i.e., greater left than right activity) coincides with approach behaviors (Mennella et al., 2017; Papousek et al., 2012). The balance between the activity of the right and left prefrontal lobes could be reflected by the alpha asymmetry in the frontal lobe (Allen et al., 2004; Mennella et al., 2017). Consequently, left-lateralized alpha asymmetry in the frontal lobe after sleep deprivation reflected an increase in withdrawal motivation and heightened vigilance for threat, contributing to increasing state anxiety.

Several limitations need to be taken into account. First, participants slept in their dormitory during the normal session, monitored by the sleep diaries and wrist actigraphy instead of the polysomnography (PSG). PSG provides a more comprehensive evaluation of sleep architecture and can identify various sleep disturbances that may interfere with the study outcomes. To enhance the accuracy and reliability of future research, it is advisable to incorporate PSG as part of the sleep assessment protocol. Second, the narrow age range of the participants (18–23 years old) might constrain the generalizability of the study’s findings. The motivational repercussions of sleep deprivation may vary across different age cohorts. Therefore, it is imperative for future research to delve into the age-specific ramifications of sleep deprivation on state anxiety and the underlying neural mechanisms. Third, despite the robust effect sizes observed for statistically significant variables, the relatively small sample size employed in this study might impede the broader applicability of the results. Finally, following 24 h of total sleep deprivation, participants exhibited pronounced excessive sleepiness, resulting in difficulty in maintaining stable vigilance during 5-min eyes-closed resting-state recordings. In the eye-closed conditions, it could not exclude intrusions of micro-sleep during the resting-state EEG recording. However, despite this, the data obtained from the eyes-closed resting condition could still offer more valid and pertinent information. Therefore, it is necessary to incorporate the analysis of the eyes-closed resting state in the future study.

## 5 Conclusion

In conclusion, sleep deprivation increased state anxiety. In addition, it heightened cortical excitability, resulting in decreased cortical communication efficiency. There was a left-lateralized frontal alpha asymmetry and increased delta-beta phase-amplitude coupling following sleep deprivation. Moreover, elevated state anxiety was linked to the overall increment in beta2 power, delta-beta2 PAC, and left-lateralized frontal alpha asymmetry.

## Data Availability

The original contributions presented in the study are included in the article/supplementary material, further inquiries can be directed to the corresponding author.
